# The Interplay of Genital Herpes with Cellular Processes: A Pathogenesis and Therapeutic Perspective

**DOI:** 10.3390/v15112195

**Published:** 2023-10-31

**Authors:** Hemant Borase, Deepak Shukla

**Affiliations:** 1 Department of Ophthalmology and Visual Sciences, University of Illinois at Chicago, Chicago, IL 60612, USA; hemantb@uic.edu; 2 Department of Microbiology and Immunology, University of Illinois at Chicago, Chicago, IL 60612, USA

**Keywords:** genital herpes, herpes simplex virus-2, vaccines, antivirals, immune response, microbiome

## Abstract

Genital herpes, primarily caused by herpes simplex virus-2 (HSV-2), remains a pressing global health concern. Its remarkable ability to intertwine with cellular processes, from harnessing host machinery for replication to subverting antiviral defenses like autophagy and programmed cell death, exemplifies the intricate interplay at the heart of its pathogenesis. While the biomedical community has extensively researched antiviral interventions, the efficiency of these strategies in managing HSV-2 remains suboptimal. Recognizing this, attention has shifted toward leveraging host cellular components to regulate HSV-2 replication and influence the cell cycle. Furthermore, innovative interventional strategies—including drug repurposing, microbivacs, connecting the host microbiome, and exploiting natural secondary metabolites—are emerging as potential game changers. This review summarizes the key steps in HSV-2 pathogenesis and newly discovered cellular interactions, presenting the latest developments in the field, highlighting existing challenges, and offering a fresh perspective on HSV-2’s pathogenesis and the potential avenues for its treatment by targeting cellular proteins and pathways.

## 1. Introduction

Herpes simplex virus (HSV), a member of the Herpesviridae family, is one of the most prevalent viral pathogens globally [1]. Historical observations dating back to Hippocrates coined the term ‘Herpes’, noting the virus’s propensity for recurrent lytic infection [1,2]. HSV is an enveloped, double-stranded DNA virus with a genome size of around 150 kilobase pairs (kbp). HSV’s genome encodes various polypeptides crucial for attachment to host cell, viral replication, lytic and lysogenic life cycles, transcriptional activation, and virulent pathogenesis [3,4]. Among the nine different types of human herpesviruses, HSV-1 and HSV-2 are the most studied, known for their broad host range, infecting neonates, adolescents, and adults [4]. Nearly the entire human population is seropositive for some type of herpesviruses, with neonates exhibiting the severest clinical outcomes when infected with HSV-2 [5,6].

HSV-2 possesses a double-stranded DNA genome with 74 genes encoding approximately 84 proteins, critical for viral component formation, infection, replication, and latency establishment [7]. Infection, neuro-invasion, and latency establishment are HSV-2’s key features [8,9,10]. Remarkably, under experimental conditions, HSV-2 demonstrates a host range beyond humans, causing diverse pathologies in various models [11,12,13].

In natural human infections, HSV-2 primarily spreads through intimate genital tract contact, resulting in persistent infection [14]. The World Health Organization (WHO) reports that 491 million sexually active individuals aged 14–49 harbor HSV-2, with an estimated 12% global prevalence of genital herpes in 2015 [15,16,17]. Approximately 400 million people are at risk of genital herpes with latent persistence, with women having a twofold higher seropositivity than men [18,19,20]. Despite antiviral therapy, HSV-2 can cause severe neurological complications and mortality, with a transmission rate of 1 in 10,000 live births [21]. Like HSV-2, HSV-1 can cause genital infection through transmission from the oral or genital area of an infected host. Recently, cases of genital herpes caused by HSV-1 have been increasing. However, genital herpes recurrences are less frequent with HSV-1 than with HSV-2 [1,2,14]. Comprehensive research in the twentieth century contributed to our understanding of HSV’s structure, function, and pathogenesis, which was crucial for preventive measures and treatments [20,22,23,24,25,26]. 

**Figure 1:**
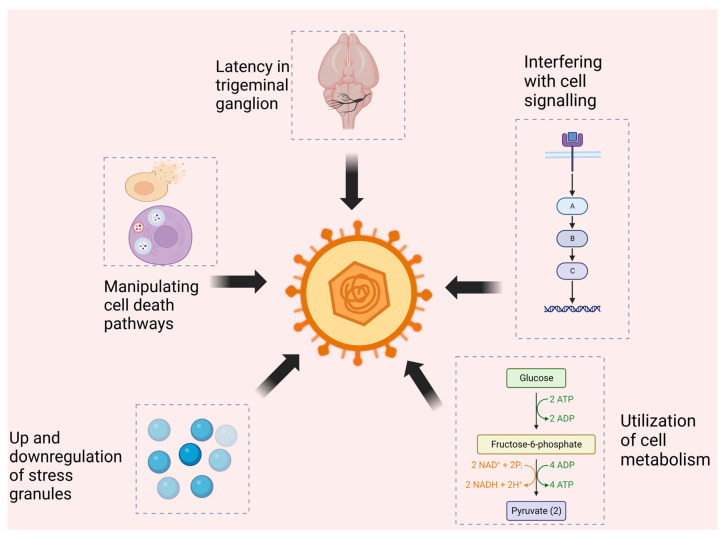
HSV-2’s survival strategies and impact on host defense mechanisms: HSV-2 employs multiple strategies to persist and replicate, including latency in the trigeminal ganglion and periodic reactivation episodes. The virus disrupts cell death pathways, notably autophagy, to its advantage. Additionally, HSV-2 has the capability to induce aberrations in cell signaling and modify metabolic pathways.

HSV-2 is a lifelong sexually and asexually transmitted infection, predominantly acquired from partners shedding the virus [27]. It ranks as the most prevalent sexually transmitted disease (STD) in the United States, targeting the skin and mucous membranes while cycling between lytic and latency phases [1,14,28]. The initial infection leads to viral replication at the exposure site and dormancy in the sacral ganglia, evading host immune responses in multiple ways [18,29] (Figure 1). HSV-2 primarily manifests as genital ulcer disease (GUD) and can cause neonatal infections, inflammation, and, less frequently, ocular infections, encephalitis, and meningitis [8,30,31,32,33,34,35]. Immunocompromised individuals and neonates face more severe HSV-2 infections due to various genetic, immune, and epigenetic factors [17,28].

The available treatments for HSV-2, including nucleoside analogs like acyclovir, valacyclovir, and famciclovir, reduce symptoms but fail to eliminate the virus [27,36,37]. HSV-2 remains latent in neuronal cells, causing recurrent infections triggered by factors like injury, infections, stress, UV exposure, and immunosuppression [1]. In the following sections, we will summarize the key steps in HSV-2 pathogenesis. As we delve deeper into understanding the intricate cellular interactions involved in HSV-2 pathogenesis, the potential for developing novel therapeutic approaches targeting cellular proteins and pathways offers hope for more effective management and treatment of this persistent global health concern.

## 2. Life Cycle of HSV-2 

The life cycle of both HSV-1 and -2 comprises several pivotal stages, which have been comprehensively discussed in many reports [1,4,26,29]. The description below of the steps involved in the viral replication is based on studies conducted on both HSV-1 and HSV-2. It commences with viral glycoproteins (gB and gC) attaching to heparan sulfate proteoglycans on cell surface protrusions, facilitating efficient access of the virions to the host cell body via a unique F-actin-dependent extracellular transport phenomenon known as ‘surfing’ [38]. Subsequently, HSV-2 enters the host cells through a fusion of the viral envelope with endocytosis, with the gD protein being instrumental in this process by interacting with cell receptors such as nectin-1 and herpesvirus entry mediator (HVEM) [39]. Once inside the cell, the viral capsid and tegument disperse in the cytoplasm. The capsid is then transported to the nucleus with the assistance of dynein, dynactin, and heat shock protein 90 [40,41]. VP22 is an important tegument protein of HSV that aids in the spread of the virus. It has been reported that the reorganization of microtubules is helpful for the nuclear transport of VP22 [42].

Within the nucleus, the regulated sequential expression of viral genes, encompassing immediate early, early, and late genes, leads to the synthesis of various proteins, including infected cell proteins and tegument proteins, all of which play pivotal roles in viral replication and the formation of viral components. Notably, early genes contribute to immune evasion and neurovirulence, while early and late proteins are essential for replication, structural components, and viral egress [43]. Finally, mature virions exit the nucleus into the cytoplasm and acquire a new envelope derived from the Golgi membrane [29,44]. Following the primary infection of keratinocyte cells in the genital region, HSV-2 spreads to nerve endings and migrates to the site of latency in the dorsal ganglia [45]. Upon reactivation, HSV-2 again travels to the genital area, leading to ulceration in the keratinocytes [14,46,47]. HSV-2 can propagate by infecting neighboring healthy cells or persist in a latent state in the dorsal ganglia [43]. Latency is an interesting survival feature of HSV-2 as the viral genome is present in a non-infectious state with potential to cause recurrent infection [43]. The role of epigenetic regulation, latency-associated transcripts (LATs), factors responsible for the establishment and reversal of latency, and drugs targeting the latent virus are some of the important aspects of HSV-2 latency [11,18].

## 3. Transmission

HSV-2 spreads through two primary modes—mother-to-fetus and sexual contact. Maternal transmission poses risks to neonates, ranging from mild to life-threatening conditions, with a heightened risk during pregnancy, particularly in cases of maternal acquisition or reactivation of the virus [2,29]. Often asymptomatic, genital herpes can go unnoticed, increasing neonatal infection risks, especially during the peripartum period, postnatally, or in utero. Factors such as vaginal delivery, invasive instruments, neonatal membrane rupture, and the presence of genital HSV-2 contribute to transmission [48,49]. Guidelines from the American College of Obstetricians and Gynecologists recommend suppressive antiviral therapy from 36 weeks of gestation and cesarean delivery for mothers with genital lesions to minimize transmission risk. However, complete elimination remains challenging, prompting ongoing research into vaccines against maternal HSV transmission, combination antiviral therapies, and antiviral agents that penetrate the blood–brain barrier [25]. Genital herpes primarily spreads through direct sexual contact with an infected partner displaying genital lesions. Asymptomatic carriers unknowingly shed the virus, with approximately 10.2% shedding during asymptomatic infections and 20.1% during symptomatic ones [50]. Behavioral and social factors, such as age, race, gender, marital status, and location, influence HSV-2 infection rates [1,51,52]. Clinical manifestations include painful genital ulcers, vesicles, blisters, and systemic symptoms like fever and headache [53]. The incubation period ranges from 2 to 12 days following exposure, with three clinical stages: primary genital herpes (without previous antibodies in the host), non-primary genital HSV (simultaneous infection of different HSVs), and recurrent genital HSV, each characterized by varying levels of reactivation and lesion formation and diagnosed through serological analysis [54].

## 4. Herpes Diagnosis: Existing and Emerging Diagnostic Tools and Techniques

**Figure 2:**
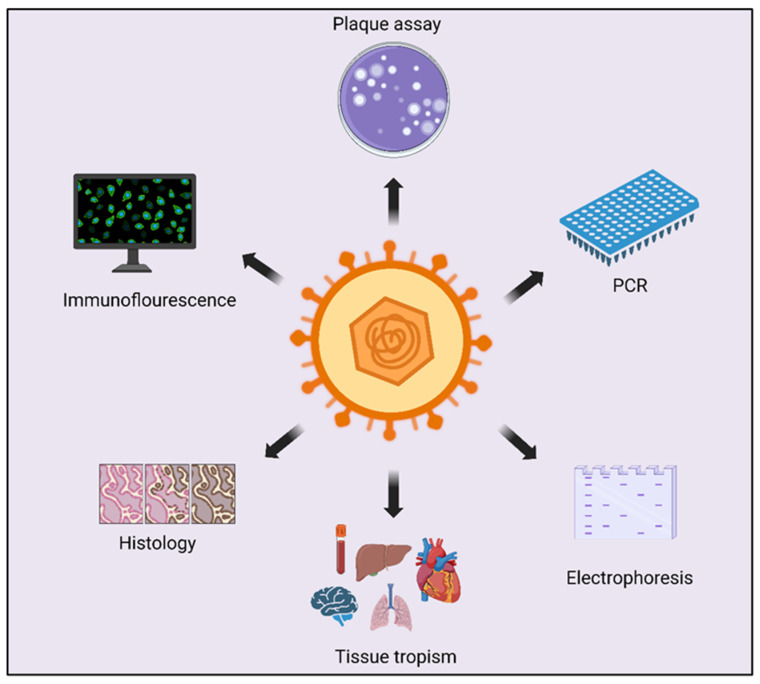
Representative diagnostic methods for investigating HSV-2 infection: Several diagnostic techniques are employed to study HSV-2 infection. The gold standard for infectious virus detection is the plaque assay. Polymerase chain reaction (PCR) is utilized to identify viral genetic material. Electrophoresis is employed to detect both cellular and viral proteins. Immunofluorescence and histochemistry serve as valuable tools for pinpointing the cellular localization of viral particles.

Diagnosing HSV-2 is crucial for disease control, treatment, and prevention. Several factors affect diagnosis, including the infection stage, sample handling, detection methods, and reporting. A variety of cell-based assays and in vivo methods have been developed to diagnose and study HSV-2 infection (Figure 2). The main approaches involve detecting the virus and its constituents (genetic material and proteins) and analyzing virus-specific antibodies in the host serum [55]. Common diagnostic methods for HSV-2 include swab collection from genital ulcers for virus culture (direct method), polymerase chain reaction (PCR) or nucleic-acid amplification assays (NAATs) for viral genetic material detection (indirect or molecular method), serological tests, and gel electrophoresis like Western blotting [56,57,58]. Traditional viral culture is time consuming and may yield false negatives, however; PCR, on the other hand, is highly sensitive, detecting lower amounts of HSV-2 genetic material [59,60]. The Tzanck smear test, which analyzes cytopathic alterations in active genital herpes lesions, lacks specificity and requires expertise [55]. The use of fluorescein-tagged monoclonal antibodies for HSV antigen detection is specific but less sensitive [50]. PCR stands out as the gold standard due to its sensitivity, specificity, and rapid results [61]. Serotype-specific analysis distinguishes HSV-2 from HSV-1 and is essential for treatment and counseling. Serological testing, using enzyme-linked immunosorbent assay (ELISA) to detect unique envelope glycoproteins (gG1 for HSV-1 and gG2 for HSV-2), is another sensitive method [62]. Point-of-care (POC) devices offer advantages like on-site, rapid, portable, and cost-effective detection with minimal expertise needed. These microfluidic devices employ various principles, including immunofluorescence, PCR, and ELISA, to name a few [55]. For instance, Laderman et al., (2008) developed a POC device for HSV-2 detection using gold nanoparticles and anti-HSV-2 IgG, showing minimal cross-reactivity [63]. Another group created a smartphone-based HSV-2 sensor using luminescent nanoparticles and recombinant glycoprotein from HSV-2, achieving high sensitivity and specificity [33].

## 5. Cell-Intrinsic Immune Response and Cell Death Pathways

The cell-intrinsic immune response plays a crucial role in controlling HSV pathogenesis. However, viruses have developed mechanisms to evade intrinsic immunity, and data from previous studies suggest that HSV-2 latency in neurons and subsequent reactivation is a result of its ability to bypass immunity [64,65,66]. Several viral proteins, such as UL41 and ICP34.5, can interact with one or more signaling molecules (interferons, TLR, NF-kB, etc.) involved in curbing viral infection and spread [67]. Interferons (IFNs) are a family of antiviral proteins that signal adjacent cells to release antiviral molecules. However, viral tegument proteins can react with IFN signaling and NF-kB transcription, rendering IFN synthesis ineffective [65]. VP24, which is required for HSV’s viral capsid, can affect IFN-B production and block IRF3 activation by stopping the interaction between Tank binding kinase 1 (TBK1) and interferon regulatory factor 3 (IRF3) during infection, leading to the suppression of IFN expression [68]. ICP34.5 can also interact with TBK1 via the TBK-1 binding domain to block IFN synthesis [69]. Therefore, the generation of an innate response by IFNs is a less effective host strategy to control HSV-2, and other signals such as tumor necrosis factor and cytokines play a crucial role in IFN stimulation by NF-kB [70,71]. Apoptosis is one of the host defense mechanisms to curtail viral infection and limit viral nuclear proliferation [72].

Stress granules (SGs) consist of mRNA and proteins and are localized in the cytoplasm, formed in response to cellular stress [73]. Depending on the viral strain, stress granule formation is either upregulated or downregulated [73,74]. Several reports highlighted that, upon HSV-2 infection of HeLa cells, UL41 hindered stress granule accumulation and provided a favorable environment for viral multiplication [75,76,77]. SGs are attracting attention owing to their role in facilitating viral replication and avoiding translational arrest [71,72,73,74,75]. In another study, HSV-2 was found to block SG accumulation in HeLa cells [75]. Virion components are found to mediate this inhibition of SGs, as HSV-2 defective in UL41 produces SGs during late infection [77]. The removal of endoribonuclease activity of the virion host shutoff (Vhs) protein creates a defect in the process of halting SG formation [77].

Multiple host cell death pathways (apoptosis, necroptosis, pyroptosis, and autophagy) constitute the cell-intrinsic defense mechanisms against viral infections, but viruses hinder infected cell death that would otherwise limit the infection [78,79]. Shrinkage and swelling of the cell, membrane blebbing and rupturing, fragmentation of genetic material, and mitochondrial permeabilization are some of the features of cell death that give opportunities to immune cells to get rid of these infected cells [79]. HSV manipulates multiple cell death pathways for its survival and replication. Caspase (cysteine proteases) is the main regulator of apoptosis and compromising caspase activity also prevents necroptosis [78]. The large subunit of ribonucleotide reductase is used by HSV to mask apoptosis via caspase 8 inhibition, whereas ICP6 and ICP10 impede receptor-interacting protein kinase 1 (RIP1) and RIP3 interaction, which are important for necroptosis [80]. Several other viral proteins such as gD, Us3, gJ, and a transcript, LAT, have also been reported to disrupt cell death pathways [78,79,80].

## 6. Cellular Metabolic Changes after HSV-2 Infection

Cellular metabolic changes after HSV-2 infection are intricately linked to the immune response and viral infection processes [81]. Various cell metabolites, including glyceraldehyde 3-phosphate dehydrogenase (GAPDH), phosphoenolpyruvate (PEP), succinate, and reactive oxygen species (ROS), interact with interferons, interleukins, T cells, and macrophages, profoundly affecting the immune response [81]. As an intracellular, obligatory parasite, HSV-2 relies entirely on the host cell’s metabolic processes to generate the biomolecules and energy necessary for viral replication. In most cases, viruses increase glycolysis and fatty acid biosynthesis to meet their energy needs for replication [81]. HSV infection disrupts oxidative phosphorylation and the Krebs cycle, resulting in the rapid metabolism of mitochondrial DNA [82]. Notably, studies have shown that HSV can enter cells in the absence of glucose but cannot produce infectious progeny, emphasizing the virus’s dependence on glucose for replication [83,84]. Additionally, research has demonstrated increased glucose uptake in Vero cells infected with HSV-1 [85].

In response to HSV-2 infection, the immune system activates interferon and various lymphocytes, such as B and T cells, to combat the virus [64,65]. However, HSV-2 has evolved strategies to evade the immune response by metabolically reducing the constituents available for the synthesis of antibodies and the production of MHC molecules, complement proteins, and NK cells [86,87]. The viral protein Vhs acts as an endoribonuclease, shutting off the host cell’s mRNA and repurposing its translational machinery for viral replication. Vhs also dampens cytokine and chemokine synthesis, leading to a less effective proinflammatory response [88,89].

## 7. HSV-2 and the Host Microbiome

**Figure 3:**
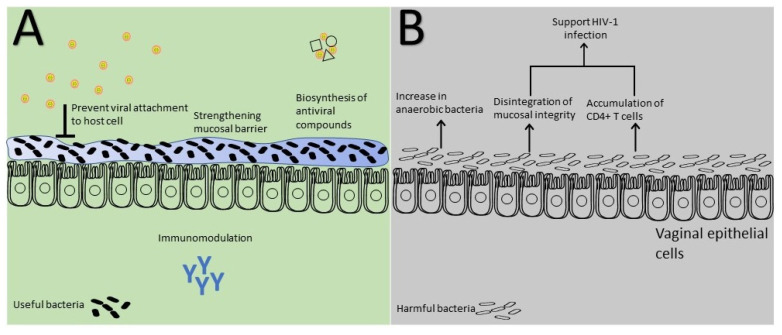
Host microbiota and its dual impact following HSV-2 infection: The host microbiota exerts beneficial effects (**A**) by masking cell receptors and enhancing entry barriers; however, it can also have detrimental consequences (**B**) by disrupting membrane integrity and promoting the proliferation of anaerobic microflora after HSV-2 infection.

The human microbiome, or microbiota, houses trillions of bacteria that play essential roles in metabolism, immunity, homeostasis, and behavior, often connected with viral infections [90,91]. Microbiome-based therapies, including probiotics, gut microbiota manipulation, and fecal microbiota treatment, can mitigate viral disease pathogenesis. In a study by Mousavi et al. (2018), *Lactobacillus crispatus* treatment before, during, and after HSV-2 infection reduced infectivity by acting as a barrier to the virus, likely trapping it or blocking HSV-2 receptors [92]. *Bacteroides fragilis* produces capsular polysaccharide A (PSA), which binds to macrophages and TLR2+ cells, increasing IL-10 secretion by regulatory T cells [93]. In a mouse model of HSV-1 infection, PSA upregulated IL-10, preventing brainstem inflammation [94] (Figure 3).

Prophylactic PSA administration protected against inflammatory neutrophils and monocyte invasion of the brainstem due to HSV-1, suggesting translational potential in combination with acyclovir against encephalitis and neurological diseases [94]. Bacterial vaginosis (BV) disrupts the vaginal microbiota by reducing beneficial bacteria (lactobacillus) and increasing anaerobic bacteria, negatively impacting reproductive health, and increasing the risk of sexually transmitted infections, including AIDS [19,20,95]. HSV-2 infection is associated with a 30% increase in BV incidence, likely due to altered vaginal microbiota caused by immune reactions on the mucosal surface [96]. Another study highlighted the close relationship between genital microbiota and HSV-2 in acquiring HIV [97]. Altered bacterial composition, including more anaerobic bacteria (*G. vaginalis*, *P. bivia*) and reduced *L. crispatus*, combined with HSV-2 infection, led to mucosal inflammation, disruption of mucosal integrity, and CD4+ T cell accumulation, supporting HIV replication [97].

## 8. Challenges in the Development of Potent Anti-HSV-2 Drugs

**Figure 4:**
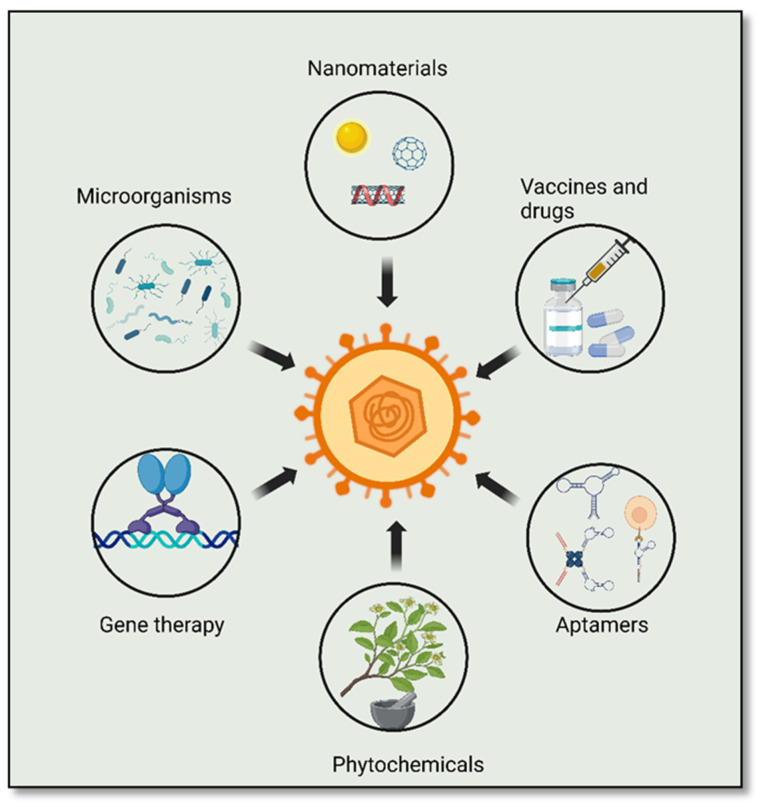
Diverse strategies for treating HSV-2 infection: Various approaches, including vaccines, drugs, nanomaterials, aptamers, and phytochemicals, have demonstrated effectiveness in preventing viral attachment, cell entry, and the release of mature virus. Additionally, gene editing can be harnessed to enhance the immune response and facilitate the development of antiviral vaccines.

There are several challenges in the development of potent antiviral therapies for the treatment of HSV-2. Additionally, many antiviral drugs have been reported to interact with other drugs, leading to toxic side effects that hinder long-term treatment [98]. The short half-life of antiviral drugs also requires frequent dosing, which can increase the risk of drug resistance and toxicity in immunocompromised patients [99]. The viral latency associated with HSV-2 infection increases the difficulty in diagnosis and subsequent treatment [100]. The selectivity of antiviral agents for virus-specific targets over host cell molecules is another challenge in antiviral therapy [101]. Most of the current treatment regimens against HSV-2 rely on targeting viral and cellular proteins to strengthen the immune response against the virus [102]. However, viral entry into host cells is a complex process, making it difficult to develop drugs that can effectively inhibit viral entry [103]. A range of local and systemic drugs including acyclovir, valacyclovir, penciclovir, famciclovir, cidofovir, foscarnet, idoxuridine, and trifluridine are currently available for the treatment of genital herpes [30,104,105,106]. However, the emergence of acyclovir-resistant strains poses a significant challenge to the effective treatment of HSV [107,108]. Although anti-HSV-2 drugs can suppress viral replication and maintain the virus in a latent stage, they cannot completely eradicate the virus or prevent frequent reactivation [109]. Additionally, these drugs can be toxic to the host and do not inhibit viral translation [110,111]. Therefore, there is an urgent need to develop safe, highly effective, novel drugs and combination synergistic therapies. These include microbicides, vaccines, as well as an emerging class of dual-acting combinations of microbicide and vaccine, termed microbivacs [112]. Drugs targeting cellular proteins involved in viral attachment, replication, latency, and proviral host factors are highly attractive for this purpose [27] (Figure 4).

## 9. Promising Treatment Strategies

### 9.1. Vaccines

Developing effective HSV-2 vaccines is crucial due to limitations in the current antiviral treatments [113,114]. Vaccine development has faced challenges due to the complex virus–host interactions and latency; however, despite multiple obstacles, vaccine candidates, including live-attenuated, killed-virus, DNA, mRNA, and subunit vaccines, are in clinical trials [115] (Table 1).

Monoclonal antibodies, adjuvants, viral antigens, aptamers, siRNAs, nanomaterials, phytochemicals, and microbial metabolites are being explored to stimulate prolonged immune responses [109]. However, vaccine candidates that induce antibody production and cytotoxic T lymphocyte responses in vitro and in rodents have shown limited protection in humans, highlighting the need for broader targets [64]. Xu et al. (2019) suggest effective vaccine strategies, including mimicking the viral replication cycle, generating innate and adaptive immune responses, eliminating viral molecules without harming host tissues, and promoting antigen presentation without compromising apoptosis [64]. A subunit vaccine called GEN-003/MM-2, containing HSV-2 glycoprotein D2 and ICP-4 in a matrix M-2, reduced genital ulcers and viral shedding in guinea pigs infected with HSV-2 [125].

Vaxfectin, a DNA-based vaccine with gD, tegument proteins, and a lipid-based adjuvant, reduced HSV-2 replication and latent HSV-2 DNA in guinea pigs [116]. Considering the link between HSV-2 and HIV, microbicides, including compounds like cellulose sulfate, SDS, zinc acetate, carrageenin, and natural products, are being explored to prevent viral entry, cell-to-cell spread, and viral membrane disruption [126]. While microbicides face challenges like dosage, interference by seminal plasma, hormonal cycles, and potential toxicity, vaccines hold significant prophylactic potential [126].

### 9.2. Gene Editing

Modern techniques are currently being developed to target HSV at the gene level. RNA interference (RNAi), zinc finger nucleases (ZFNs), transcription activator-like effector nucleases (TALENs), antisense oligonucleotides, and clustered regularly interspaced short palindromic repeat (CRISPR)-associated protein 9 (Cas-9) have shown potential for editing the viral genome to develop more-effective and less-toxic antiviral therapies with an effective host immune response and lower chances of generating mutations [127]. Studies have shown that CRISPR/Cas9-mediated disruption of viral genes involved in DNA replication, tegument proteins, and capsid proteins can cause malfunctioning of HSV replication in Vero cells [128,129]. Knockdown of vital genes involved in the lytic cycle, such as ICP4, by siRNA resulted in decreased ICP4 expression in retinal epithelial cells, human trabecular meshwork cells, and Vero cells, with inhibition of cytopathic effects [130]. However, the major bottlenecks of gene editing technology are possible CRISPR/Cas9 mutant generation, low transfection rates, and toxicity associated with transcription-facilitating reagents [131].

### 9.3. Antiviral Therapies Targeting Cellular Proteins

Multiple reports have clearly suggested a decrease in potency and the high toxicity of currently available anti-HSV drugs due to the emergence of resistant strains, making the development of newer treatments crucial [5,132]. Hopkins et al. (2020) reported the anti-HSV-2 potential of a small molecule known as BX795 [27]. BX795 exhibited in vitro antiviral activity at 10 µM in vaginal keratinocyte cells infected with HSV-2. Further, BX795 was found to be effective at a 50 µM concentration in an in vivo murine genital infection model. More interestingly, the vaginal keratinocyte cells can tolerate high concentrations (80 µM) of BX795, suggesting its cytocompatibility. BX795 inhibits HSV-2 by inhibiting the phosphorylation of protein kinase B (AKT). Cells infected with HSV-2 at a 0.1 multiplicity of infection (MOI) followed by treatment with BX795 (10 µM) showed significantly lowered viral (ICP27, gD) transcript levels, and the same was observed in a Western blot. In conclusion, BX795 stops the viral cellular translational machinery, thereby inhibiting viral multiplication. Based on earlier results, the author further proved that therapeutic prophylactic treatment of vaginal keratinocytes with BX795 strongly prevents future HSV-2 infection. In a quest to further the understanding of the mechanism of action of BX795, in vitro, in vivo, and in silico docking studies revealed that BX795 lowers AKT phosphorylation, along with its downstream targets, 4EBP1 and p70S6K [133].

The antiviral potential of highly porous activated carbon (HPAC) against HSV-2 was recently demonstrated in both in vitro and in vivo models [134,135]. In vitro studies have shown that activated charcoal can trap virions and prevent infection. Interestingly, loading acyclovir into activated charcoal (DECON—drug encapsulated carbon) inhibits HSV-2 at very low concentrations (IC50 of 0.08 mg/mL) and is able to restrict HSV-1, pseudorabies virus (PRV), and bovine herpesvirus (BHV). In a murine model of genital infection, the combination of highly porous activated carbon (HPAC) loaded with acyclovir, known as DECON, greatly enhanced the efficacy of acyclovir with lower dose requirements and a shorter treatment time. Furthermore, DECON possesses beneficial characteristics such as a prophylactic and sustained drug release platform that is biocompatible, cost efficient, and immunologically neutral [135]. Additionally, 4-phenylbutyrate (PBA), a compound commonly used to treat urea cycle disorders, was found to have anti-HSV activity and synergistic potential when used in combination with trifluridine (TFT). PBA was shown to mask the regulation of the viral-supporting host protein CREB3, resulting in the inhibition of viral translation. The combination of PBA and TFT lowered the concentration of TFT required for antiviral activity [136].

### 9.4. Nanomaterial: Inhibitors of Proviral Functions of Cellular Proteins

Nanomaterials with the capability to interfere with the proviral functions of cellular proteins find applications in the detection, diagnosis, and treatment of viral diseases [106,137,138]. This emerging field harnesses a wide range of nanomaterials, including carbon dots, graphene, polymeric nanomaterials, silver nanoparticles, gold nanoparticles, and others, to inhibit mechanisms that facilitate viral infections, ushering in new possibilities in drug discovery and targeted drug delivery [139,140,141]. These nanomaterials possess unique properties, such as size, shape, composition, charge, and surface functionalization, which can be strategically employed to either detect or competitively inhibit various cellular processes crucial for viral infections [138]. Nanomaterials are the next generation of antimicrobials, capable of disrupting multiple stages of viral infection, ranging from attachment and entry to replication, egress, and latency, all while modulating inflammation and the host immune response [112,140]. Nanomaterials can be engineered to disrupt viral capsids, mechanically damage viruses, enhance targeting precision, and even mimic host receptors [142].

**Figure 5:**
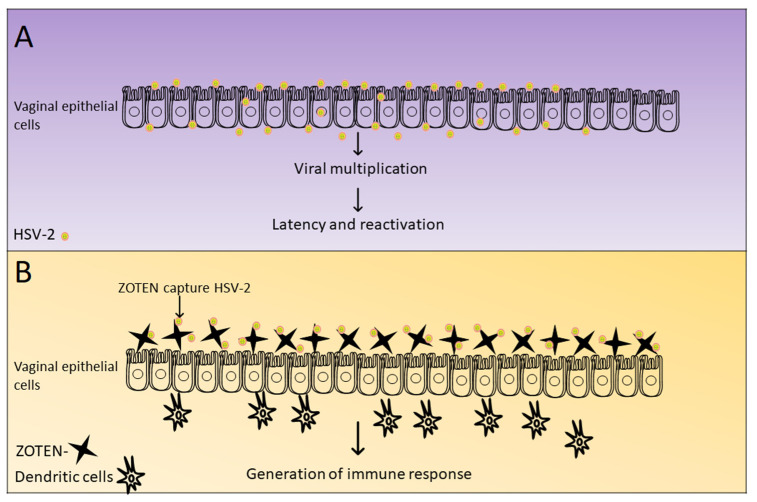
Microbivac action: (**A**) HSV-2 infection in vaginal epithelial cells: Results in viral multiplication and disease progression. (**B**) ZOTENs as a microbivac against HSV-2: Functions by trapping the virus, leading to a substantial decrease in disease severity, enhanced antigen presentation, and an improved mucosal immune response.

For instance, silver nanoparticles (AgNPs), known for their diverse properties, have demonstrated inhibitory effects against a spectrum of viruses, including HIV-1, H1N1, PPRV, vaccinia virus, and poliovirus [140]. Gold nanoparticles (AuNPs), appreciated for their biocompatibility, effectively blocked HSV-2G, showcasing their potential as viral entry inhibitors [143]. Zinc oxide tetrapod nanoparticles (ZOTENs), intentionally designed for efficient virus capture, have exhibited microbicidal potential against genital HSV-2 strain 333, resulting in reduced viral shedding and inflammation. The captured virions are utilized for antigen presentation and immune system development. Therefore, ZOTENs are considered an effective microbivac that functions both as an effective microbicide and an antigenic platform for lasting immunity against the virus [112,144] (Figure 5). Similarly, carbon nanodots (C-dots) functionalized with amine and boronic acid have displayed high efficacy at low concentrations, effectively inhibiting HSV-1 entry without causing cytotoxic effects [102]. The examples above underscore the promising role of nanomaterials in combating viral diseases, offering innovative approaches to detection, prevention, and treatment by disrupting cellular processes that facilitate viral replication and infection.

### 9.5. Natural Compounds

Due to the increasing resistance to widely prescribed anti-HSV drugs, including acyclovir, there is a growing demand for more-potent compounds from natural sources, such as plants and microorganisms. The major classes of antiviral compounds from natural sources include alkaloids, flavonoids, terpenes, polysaccharides, phenolic acids, peptides, and steroids [145,146]. Diverse compounds of natural origin may be highly potent against HSV due to their multiple inhibitory mechanisms, making antiviral resistance difficult for viruses [147] (Table 2).

### 9.6. Aptamers

Aptamers are either oligonucleotide or peptide molecules with a high affinity to bind a diversity of biological important molecules and are proposed as an alternative to antibodies for virus and viral antigen detection [156,157]. Aptamers can not only prevent viral entry inside the host cell but can also act on other vital processes of the virus, such as transcription and translation [156]. The above characteristics demand more research on aptamers with respect to their usefulness in the therapy and diagnosis of viral diseases [158]. Moore et al. (2011) fabricated RNA aptamers through the SELEX process and tested their potential to neutralize HSV-2. They found that the aptamers inhibited HSV-2 infection with a low IC50 range (20–50 nM) by reacting with gD and inhibiting the HVEM and nectin 1 infection pathway [159].

## 10. Optineurin and Autophagy in HSV-2 Infection

Research into controlling HSV-2 infection has recently turned its attention to understanding the pivotal cellular factors that either amplify or suppress viral pathogenesis. This could be a game changer in developing new therapeutic strategies against HSV-2 [136]. A notable protein, optineurin (OPTN), previously recognized for restricting Salmonella enterica infection has emerged as a significant player in the control of HSV-2 pathogenesis [160]. Patil et al. (2022) found that cells without OPTN (OPTN−/−) exhibited a more pronounced HSV-2 infection compared to their OPTN-containing (OPTN+/+) counterparts [161]. This disparity was largely attributed to OPTN’s crucial role in modulating protective autophagy and CCL5 levels. Supporting these findings, a variety of investigative techniques were employed, which demonstrated a striking fivefold HSV-2 increase in OPTN−/− cells compared to the controls. A deeper dive into the study’s data revealed a noteworthy uptick in the conversion of the protein LC3-I to LC3-II in OPTN+/+ cells during HSV-2 invasion, a recognized marker of autophagy [161,162].

Autophagy, a cell recycling system, plays a pivotal role in discarding unwanted cellular components, hence acting as a lifeline during cellular stress scenarios [163]. The dynamics of autophagy in the context of HSV-2 remained ambiguous until Yakoub and Shukla (2015) demonstrated that HSV-2 maintains autophagy at a baseline level, rather than enhancing it [164]. When autophagy was pharmacologically hindered (chloroquine treatment), there was a marked decline in HSV-2 virulence [164]. The inhibition of interferon β by ICP22 and ICP27 of HSV-2 pointed out the interplay between innate immunity and autophagy and viral infection [165]. A study focused on patients with autophagy-related protein mutations indicated a surge in viral replication and subsequent cell demise [166]. Conversely, reintroducing these proteins effectively curtailed these adverse effects.

## 11. Heparanase in the Pathogenesis of HSV-2

The dynamics of how cellular proteins and their corresponding regulators foster HSV-2 in exiting target cells remains largely underexplored [26,167]. Recently, heparanase (HPSE) has garnered attention for its potential involvement in the pathogenesis of HSV [168]. In a pivotal study by Hopkins et al. (2018), two critical cellular enzymes, HPSE and cathepsin L, were found to play a role in HSV-2 infection [169]. Specifically, HSV-2 infection was observed to instigate the shedding of heparan sulfate, due to a surge in HPSE levels. Meanwhile, cathepsin L has emerged as instrumental, transforming the dormant HPSE into its active state. This claim was further solidified when the application of an HPSE inhibitor markedly diminished the release of HSV-2 from vaginal cells. Delving deeper into the molecular intricacies, the escalation in HPSE after infection appears to be mediated by NF-kB. The significance of cathepsin L in HSV-2’s release and subsequent spread was accentuated when using cathepsin L inhibitor IV, which showed a reduced rate of viral egress. Beyond the realm of HSV-2, it is worth noting that HPSE’s influence extends to various inflammatory ailments, encompassing conditions like colitis, lung injuries, and other sexually transmitted diseases [170].

## 12. Conclusions

To summarize, while our understanding of HSVs has spanned a considerable duration, a definitive therapeutic solution that entirely eradicates the virus and bestows comprehensive immunity against the infection remains elusive. Nevertheless, the accelerated pace at which we are uncovering new insights concerning the HSV-2 life cycle within its host, coupled with the host’s defensive responses, paints a hopeful picture. The horizon now shimmers with the potential for achieving full-scale, multitargeted, and more effective treatment of this persistent infectious agent. A multitude of strategies are being explored to counter the menace of this virus. These include the development of synergistic medications, the repurposing of existing drugs, and innovations in vaccines, microbicides, and biological metabolites. Rapid diagnostic tools, when coupled with extensive public awareness campaigns, can also play a pivotal role in curtailing the spread and impact of the disease. It is crucial to remember that alleviating the burden of HSV-2 is not solely about countering one ailment. Effective strategies against HSV-2 can cascade into reductions in the prevalence of associated complications and co-infections, including but not limited to AIDS, various cancers, and other sexually transmitted diseases. As we move forward, a deeper dive into the realm of the host’s immune-metabolic changes and the microbiome post-HSV-2 infection is anticipated to be a goldmine of information. These insights can be harnessed to forge robust defenses and therapeutic interventions against the virus in future endeavors.

## Figures and Tables

**Table 1:** Representative vaccine candidates against HSV-2.

Type of Vaccine	Name	Description	Reference
DNA based	Vaxfectin^R^	A plasmid DNA vaccine consisting of HSV-2 gD2/UL46/UL47 genes, a cationic lipid adjuvant. In a guinea pig model, this vaccine reduces viral replication and latent HSV-2 DNA. It has prophylactic and therapeutic benefits.	[116]
DNA based	Codon mixture vaccine	A mixture of codons having ubiquitinated and non-ubiquitinated gD2, producing a cellular and humoral immune response. In a mouse model, it demonstrates a high survival rate and a low viral DNA load in the DRG.	[117]
Live attenuated	HSV-2ΔgD2	HSV-2 virus deleted in gD. Protects C57BL/6 mice from intravaginal, neuronal disease and functions via Fc receptors.	[118]
Live attenuated	HSV-2ΔNLS	An HSV-2 ICP0 virus that is 100 times more effective than the gD subunit vaccine against genital herpes.	[119]
Subunit	HSV-2 gD	Truncated gD from HSV-2 with alum as an adjuvant and 3-0-deacylated monophosporyl lipid A. Provides protection from HSV-1 but not against HSV-2. Shows only 20% efficacy for HSV-2.	[120]
Subunit	HSV-2 gB/gD/MF59	Vaccine is less effective in decreasing the rate of genital HSV-2 acquisition. High levels of neutralizing antibodies after vaccination are unable to protect from HSV-2 infection.	[121]
Defective in replication	HSV-2 529	Deletions in ULS and UL29. Immunization produces HSV-2-neutralizing antibodies in mice and guinea pigs. The vaccine effectively reduces HSV-2 infection by lowering mortality, genital ulcer severity, and shedding and warrants further study on humans for prophylactic purposes.	[122]
Peptide	AG-707	Consists of 32 HSV-2 peptides with HSP70 chaperons and saponin as adjuvant. Induces a T cell response. Prophylactic use significantly protects in a mouse model and shows activity in guinea pigs.	[123]
Peptide	HSV-2-CD8+ T cell peptide	The peptide epitope consists of a palmitic acid moiety and is an agonist of toll-like receptor 2 and generates HSV-2-specific CD8+ T cytotoxic T cells. TLR-2 mice develop less CD8+ T cytotoxic T cells, exhibiting rapid death and disease progress compared to wild-type immunized mice.	[124]

DRG, dorsal root ganglia, Vaxfectin^R^- Registered trademark.

**Table 2:** Plant secondary metabolites as anti-HSV-2 agents.

Plant Species	Bioactive Compound(s)	Major Findings	Reference
*Mallotus peltatus*	Ursolic acid	Inhibits the early stage of viral spread and multiplication (HSV-1 and -2), inhibits plaque formation and has synergistic antiviral activity with acyclovir. PCR study showed no viral DNA amplification.	[146]
*Caesalpinia* *pulcherrima*	Quercetin	Inhibits HSV-1 KOS, HSV-2 (196), and adenoviruses (ADV-3, ADV-8, ADV-11) in a dose-dependent manner by preventing early-stage multiplication in a human skin basal cell carcinoma cell line.	[145]
*Houttuynia cordata*	Quercetin	Inhibits HSV-1F, HSV-2G, and acyclovir-resistant HSV-1 by preventing viral binding and penetration. Suppresses NF-κB activation. Apart from Vero cells, other cell lines (human epithelial carcinoma cells HEp-2 and A549) were also protected from HSV infection.	[148]
*Salvia fruticosa*	Isoborneol	Inhibits glycosylation of HSV-1 gB and gD after treatment with Isoborneol.	[149]
Usnea, Psoroma, and Alectoria (lichen species).	Psoromic acid	Inhibits HSV DNA polymerase and protease. The combination of Psoromic acid with acyclovir leads to potent inhibitory action.	[150]
*Azadirachta indica*	Consortium of protein, polysaccharides, and alkaloids	Pre-incubation of aqueous extract (50–100 µg/mL) with HSV-1 blocks HSV-1 entry in target cells (RPE, Hela, CHO-KI). Inhibition of HSV-1 glycoprotein-mediated cell-to-cell fusion and polykaryocyte formation. Activity was promising against different HSV-1 strains.	[151]
*Galla* *chinensis*	Gallic acid	Acts on both HSV-1 KOS and HSV-2 (333). Impedes the expression of viral proteins (VP5, ICP2, gC, gD).	[152]
*Lepechinia speciosa*	Verbascoside	Acts against clinical isolates of HSV-2 by preventing different stages of viral replication (attachment, adsorption, and penetration).	[153]
*Terminalia arjuna*	Casuarinin	Effective in inhibiting HSV-2 12 h after infection. Disturbs late events of infection apart from preventing penetration and attachment.	[154]
*Peganum harmala*	Harmine	Inhibitor of tyrosine phosphorylation kinase. Downregulation of MAPK and NF-κB pathways.	[155]

RPE, retinal pigment epithelial; CHO, Chinese hamster ovary.
